# Pinniped (Carnivora, Phocidae) occurrences in the Azores Archipelago (NE Atlantic)

**DOI:** 10.3897/BDJ.10.e96342

**Published:** 2022-11-08

**Authors:** Luís M.D. Barcelos, João Pedro Barreiros

**Affiliations:** 1 cE3c- Centre for Ecology, Evolution and Environmental Changes, Azorean Biodiversity Group, CHANGE – Global Change and Sustainability Institute, School of Agrarian and Environmental Sciences, University of the Azores, Rua Capitão João d´Ávila, Pico da Urze, Angra do Heroísmo, 9700-042, Terceira, Azores, Portugal cE3c- Centre for Ecology, Evolution and Environmental Changes, Azorean Biodiversity Group, CHANGE – Global Change and Sustainability Institute, School of Agrarian and Environmental Sciences, University of the Azores Rua Capitão João d´Ávila, Pico da Urze, Angra do Heroísmo, 9700-042, Terceira, Azores Portugal; 2 IUCN - International Union for the Conservation of Nature, Groupers and Wrasses Specialist Group, Hong Kong, China IUCN - International Union for the Conservation of Nature, Groupers and Wrasses Specialist Group Hong Kong China

**Keywords:** Pinnipeds, regional inventory, species list

## Abstract

**Background:**

The last Pinniped species update was in 2010, as part of the list of the terrestrial and marine biota from the Azores. This list includes a chapter dedicated to marine mammals, based on previously published bibliography.

**New information:**

No new species were added since that list was publlished. However, there were new occurrences since the last update.

## Introduction

**Figure 1.**
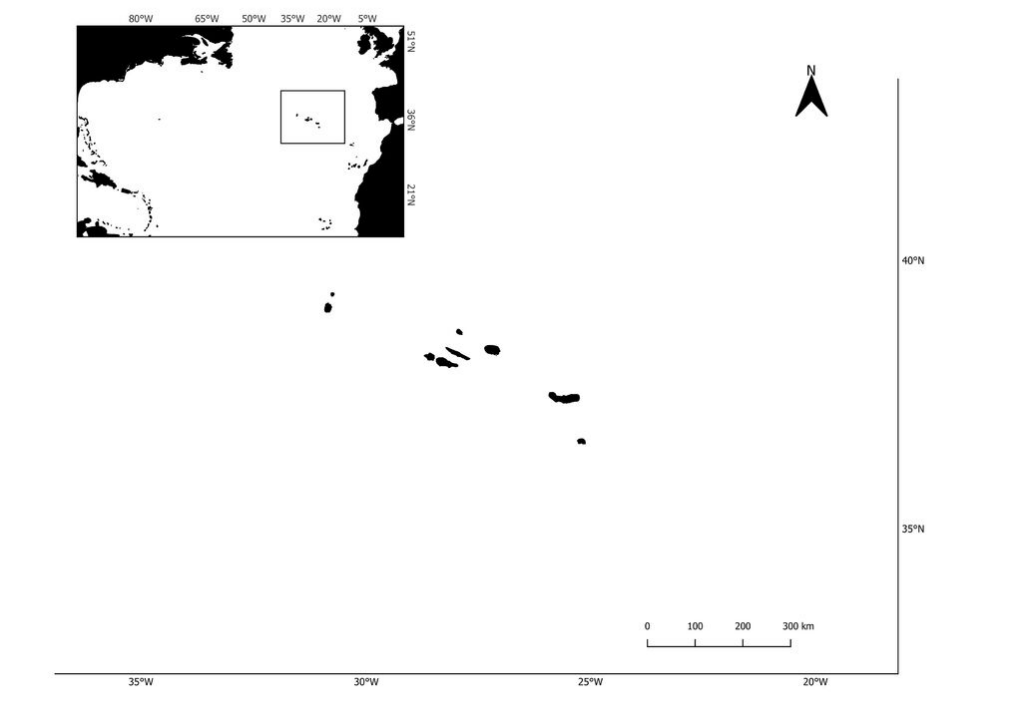
Location of Azores Archipelago.

**Figure 2.**
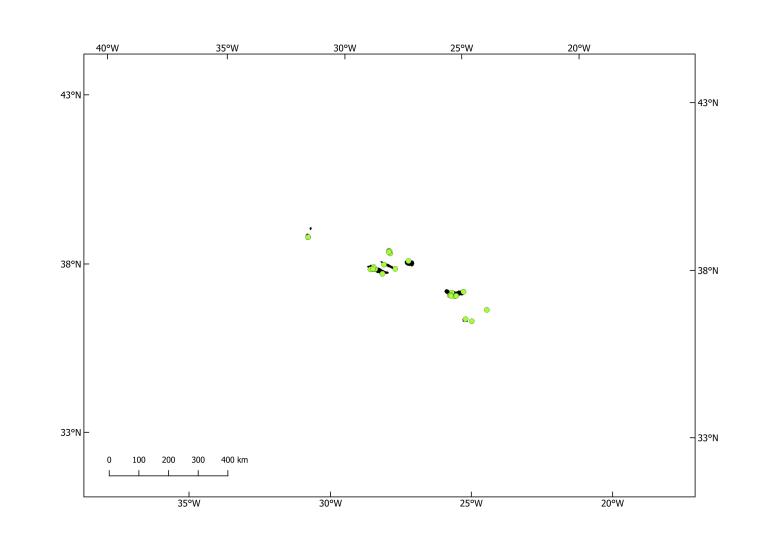
Distributions of the Phocidae species sightings within Azores Archipelago.

**Figure 3.**
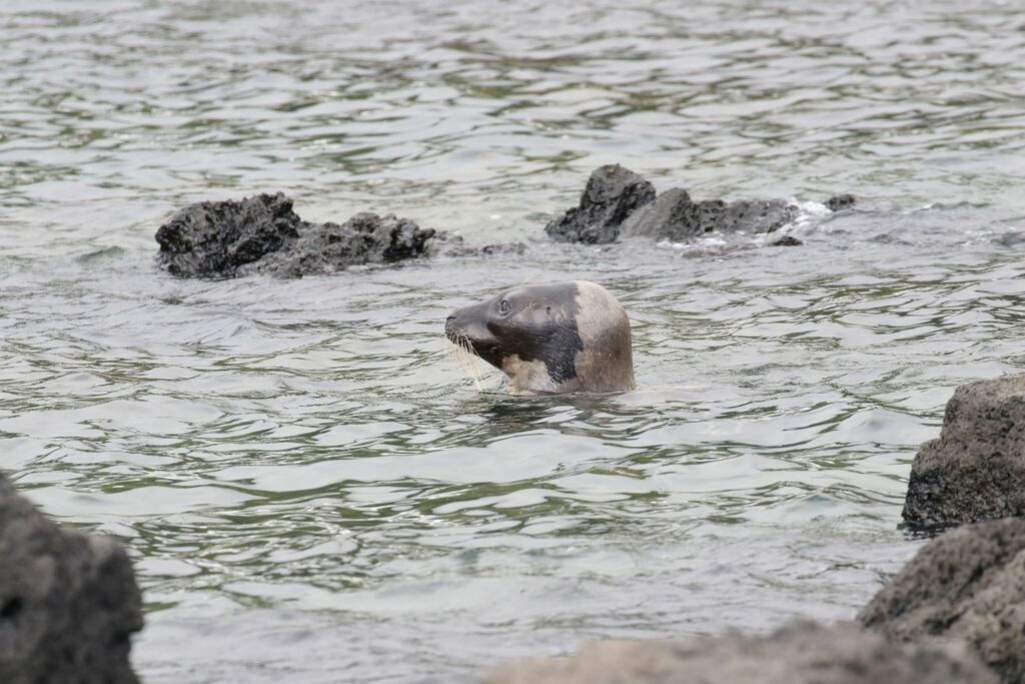
Harp seal *Pagophilusgroenlandicus* (Erxleben, 1777) on 23 August 2020 on Lajes do Pico (photo by Olivier Coucelos).

**Figure 4.**
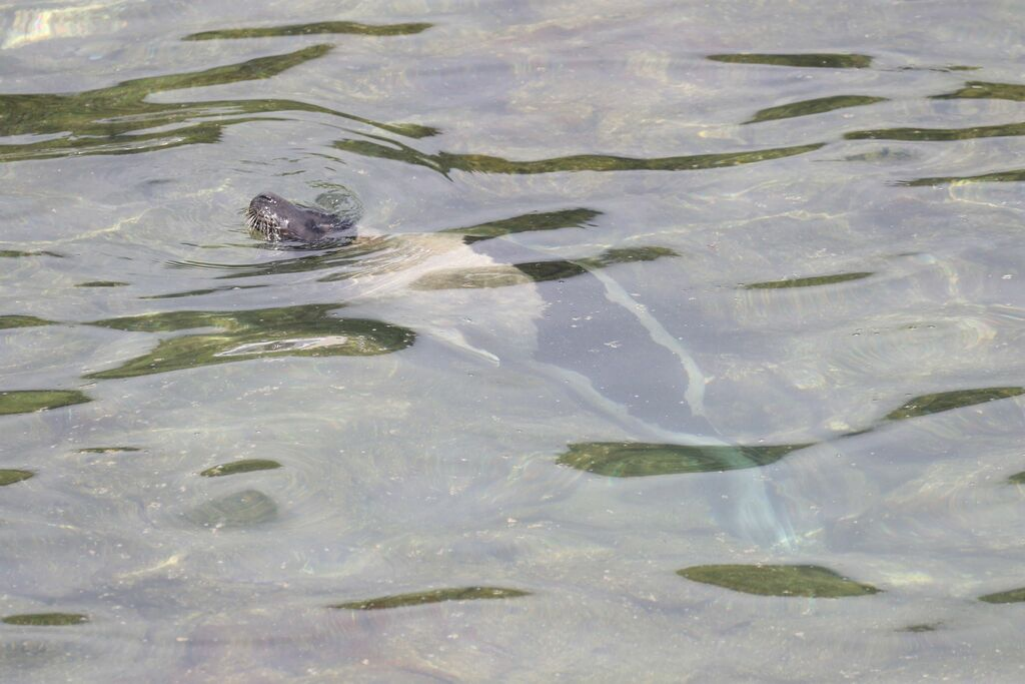
Harp Seal *Pagophilusgroenlandicus* (Erxleben, 1777) on 23 August 2020 on Lajes do Pico (photo by Olivier Coucelos).

The last list of pinniped occurrences in the Azores (Fig. 1) dates back to 2010 (Prieto and Silva 2010). Pinniped confirmed sightings are shown (Fig. 2). Since then, no new species were recorded for the Archipelago, but there were sporadic occurrences, mostly from grey seals, *Halichoerusgrypus* (Fabricius, 1791) in 2011, 2012 and 2017. The most recently confirmed sighting was a Harp Seal *Pagophilusgroenlandicus* (Erxleben, 1777), in August 2020 (Figs 3, 4), that appeared on Faial and Pico Islands. This summer vagrant is an unusual occurrence and the individual died after a week from first being seen.

After the extinction of the Azorean Monk seal colonies, *Monachusmonachus* (Hermann, 1779), species that is classified by the IUCN (International Union for Conservation of Nature Red List of Threatened Species) as endangered (see Table 1), in the early 17^th^ century (see discussion in Silva et al. 2009), all seal occurrences in the Azores are from arctic and subarctic species, although a few reports raise the possibility that Monk seals travelling from Madeira to be occasional vagrants in these waters, due to the central location of the Archipelago in the Northeast Atlantic Ocean (Fig. 1).

## General description

### Purpose

Consolidation and updating of Pinniped records in the Azores Archipelago, through the publication of the species list in GBIF (Barcelos and Barreiros 2022b), accompanied by the information available on these occurrence records.

These records are from previously published data (Silva et al. 2009, Prieto and Silva 2010) and from unpublished data provided by RACA - Rede de Arrojamento de Cetáceos dos Açores.

## Project description

### Title

AZORESBIOPORTAL - PORBIOTA

### Study area description

Azores Archipelago, including EEZ

### Design description

The Azorean Biodiversity Portal E-Infrastructure (https://azoresbioportal.uac.pt/pt/) was approved by FCT for the National Research Infrastructure in the Roadmap. The approval of Azorean Biodiversity Portal by the Portuguese E-Infrastructure Roadmap, guaranteed financial support between 2019 and 2021 and the improvement of the Portal and new products. This is quite an important achievement for this regional Biodiversity Portal. The Azorean Biodiversity Portal (ABP) is a key e-infrastructure for the integrated management of biodiversity data of the Azores, providing a large number of specialised services supporting research, policy and education (Borges et al. 2010). The evaluators considered that the submitted proposal lists some significant policy integration opportunities with the Azorean government, using the portal as part of its conservation activities for protected areas, as well as for the sustainable management of biodiversity relating to agriculture, forestry and fisheries. This was the first Biodiversity Portal in Portugal, starting in 2008 and the only one which provides easy access to island biodiversity data (Borges et al. 2010). ABP is currently recognised as a valuable outreach, management and conservation tool for all who work in science and protection of biodiversity. The large number of visits per day, the numerous international scientific collaborations, resulting in publications and academic theses and the connection with other prestigious databases demonstrate the Portal’s scientific quality, as well as its general appeal. This project initiated in 2008 under the leadership of researchers from the Azorean Biodiversity Group (CITA_A; currently cE3c -Azorean Biodiversity Group), based in the formerly Dept. of Agrarian Sciences (currently School of Agrarian & Environmental Sciences) in Terceira Island and included also the collaboration with researchers from the CIBIO-Azores, based in the formerly Dept. of Biology of the Univ. of Azores (currently School of Sciences & Technology) and more recently researchers from OKEANUS-DOP in Horta. At this moment, the Portal is being funded by the Azorean Science Ministry (Azores PO 2020 - ACORES-01-0145-FEDER-000072). The main ABP action lines are to: - improve the informatics system of the e-infrastructure to allow complex queries and improve user-friendliness - guarantee a rigorous classification for every species, providing updated comprehensive checklists, ensuring accuracy on the compilation of biogeographical information; this is the backbone of the Portal and all its products and services - provide innovative biodiversity analytical tools for both researchers and community members and invite them to contribute data to the Portal, establishing effective science communication.

### Funding

Funding Institutions: AZORESBIOPORTAL – PORBIOTA (Azores PO 2020 - ACORES-01-0145-FEDER-000072) TOTAL BUDGET: €299,901.83 EU Support: €254, 916.56. This project was financed by FEDER in 85% and by Azorean Public funds by 15% through the Operational Programme Azores 2020. This work is also funded by FEDER funds through the COMPETE 2020 Programme and National Funds through FCT - Portuguese Foundation for Science and Technology under the Research Infrastructure PORBIOTA - Portuguese E-Infrastructure for Information and Research on Biodiversity, project number POCI-01-0145-FEDER-022127.

For the period 2022-2023- Portal da Biodiversidade dos Açores (2022-2023) - PO Azores Project - M1.1.A/INFRAEST CIENT/001/2022.

Open access will be supported by the project FCT-UIDB/00329/2020-2024 (Thematic Line 1 – integrated ecological assessment of environmental change on biodiversity).

## Sampling methods

### Study extent

Azores EEZ

### Sampling description

Sightings of live or stranded animals in shore or coastal areas of the Azores Archipelago.

### Step description

Taxonomic identification and records for all available information.

## Geographic coverage

### Description

Azores EEZ

### Coordinates

33.6536 and 43.1598 Latitude; -35.4936 and -20.4584 Longitude.

## Taxonomic coverage

### Description

Taxonomic range of Pinipeds with confirmed occurrences in Azorean waters.

### Taxa included

**Table:** 

Rank	Scientific Name	Common Name
kingdom	Animalia	Animals
phylum	Chordata	
class	Mammalia	
order	Carnivora	
suborder	Pinnipedia	
family	Phocidae	
subfamily	Phocinae	
subfamily	Monachinae	
genus	*Pusa*	
genus	*Phoca*	
genus	*Pagophilus*	
genus	*Halichoerus*	
genus	*Cystophora*	
genus	*Monachus*	

## Temporal coverage

### Notes

All available records between 1970 and 2022.

## Usage licence

### Usage licence

Creative Commons Public Domain Waiver (CC-Zero)

## Data resources

### Data package title

Phocidae species in Azores Archipelago.

### Resource link

http://ipt.gbif.pt/ipt/resource?r=phocidae_species_in_azores_archipelago

### Alternative identifiers

http://ipt.gbif.pt/ipt/resource?r=phocidae_species_in_azores_archipelago

### Number of data sets

2

### Data set 1.

#### Data set name

Phocidae species in Azores Archipelago.

#### Data format

Darwin Core Archive format

#### Character set

UTF-8

#### Download URL

http://ipt.gbif.pt/ipt/archive.do?r=phocidae_species_in_azores_archipelago&v=1.7

#### Data format version

1.7

#### Description

The dates and places of occurrence of these species can be found in Suppl. material 1. The Metadata can be also consulted at Barcelos and Barreiros (2022b).

**Data set 1.** 

Column label	Column description
id	Identifier.
taxonID	Identifier of the Taxon.
parentNameUsageID	An identifier for the name usage of the direct, most proximate higher-rank parent taxon of the scientificName.
scientificName	The full scientific name, with authorship and date information.
parentNameUsage	The name of the direct, most proximate higher-rank parent taxon.
kingdom	kingdom.
phylum	phylum.
class	class.
order	order.
family	family.
subfamily	subfamily.
genus	genus.
specificEpithet	The name of the first or species epithet of the scientificName.
taxonRank	The taxonomic rank of the most specific name in the scientificName.
scientificNameAuthorship	The authorship information for the scientificName formatted according to the conventions of the applicable nomenclaturalCode.
bibliographicCitation	A bibliographic reference for the resource.

### Data set 2.

#### Data set name

Occurrences of pinnipeds (Carnivora, Phocidae) in the Azores Archipelago (Portugal).

#### Data format

Darwin Core Archive format

#### Character set

UTF-8

#### Download URL

http://ipt.gbif.pt/ipt/resource?r=phocidae_azores

#### Data format version

1.5

#### Description

An inventory of historical and actual occurrences of pinnipeds in the Azores Archipelago (Barcelos and Barreiros 2022a). The data used come from Silva et al. (2009) and from RACA - Rede de Arrojamentos de Cetáceos dos Açores (RACA-DRAM-RAA).

**Data set 2.** 

Column label	Column description
occurrenceID	Identifier of the occurrence, unique for the dataset.
basisOfRecord	The nature of the related resource.
eventDate	Date information for the occurrence.
scientificName	full name, with authorship and date information.
kingdom	kingdom.
phylum	phylum.
class	class.
order	order.
family	family.
subfamily	subfamily.
genus	genus.
specificEpithet	The name of the first or species epithet of the scientificName.
scientificNameAuthorship	The authorship information for the scientificName.
taxonRank	The taxonomic rank of the most specific name in the scientificName.
decimalLongitude	The geographic longitude (in decimal degrees). Positive values are east of the Greenwich Meridian, negative values are west of it.
decimalLatitude	The geographic latitude (in decimal degrees). Positive values are north of the Equator, negative values are south of it.
geodeticDatum	The ellipsoid, geodetic datum or spatial reference system (SRS), upon which the geographic coordinates given in decimalLatitude and decimalLongitude are based.
country	The name of the country or major administrative unit, in which the Location occurs.
island	The name of the island on or near which the Location occurs.
locality	The specific description of the place.
establishmentMeans	The process by which the biological individual(s) represented in the Occurrence became established at the location.

## Additional information

The data were provided by Rede de Arrojamento de Cetáceos dos Açores (RACA-DRAM-RAA).

## Supplementary Material

7CF6A037-5BCC-5E1B-8944-646EFE22F9A7 10.3897/BDJ.10.e96342.suppl1 Supplementary material 1 Pinniped occurrence in the Azores Archipelago (NE Atlantic) Data type occurrence Brief description Occurrence locations, dates and species' taxonomy. File: oo_757398.csv https://binary.pensoft.net/file/757398 Luís MD Barcelos & João Pedro Barreiros

## Figures and Tables

**Table 1.** Table 1 - IUCN Red List (International Union for Conservation of Nature Red List of Threatened Species) classification of the species.

**Scientific Name**	**IUCN classification**	**IUCN classification Source**
*Monachusmonachus* (Hermann, 1779)	endangered	Karamanlidis A & Dendrinos P (2015). Monachusmonachus (errata version published in 2017). The IUCN Red List of Threatened Species (2015): e.T13653A117647375. https://dx.doi.org/10.2305/IUCN.UK.2015-4.RLTS.T13653A45227543.en. Accessed on 06 October 2022.
*Pusahispida* (Schreber, 1775)	least concern	Lowry L (2016). Pusahispida. The IUCN Red List of Threatened Species (2016): e.T41672A45231341. https://dx.doi.org/10.2305/IUCN.UK.2016-1.RLTS.T41672A45231341.en. Accessed on 06 October 2022.
*Phocavitulina* Linnaeus, 1758	least concern	Lowry L (2016). Phocavitulina. The IUCN Red List of Threatened Species (2016): e.T17013A45229114. https://dx.doi.org/10.2305/IUCN.UK.2016-1.RLTS.T17013A45229114.en. Accessed on 06 October 2022.
*Pagophilusgroenlandicus* (Erxleben, 1777)	least concern	Kovacs KM (2015). Pagophilusgroenlandicus. The IUCN Red List of Threatened Species (2015): e.T41671A45231087. https://dx.doi.org/10.2305/IUCN.UK.2015-4.RLTS.T41671A45231087.en. Accessed on 06 October 2022.
*Cystophoracristata* (Erxleben, 1777)	vulnerable	Kovacs KM (2016). Cystophoracristata. The IUCN Red List of Threatened Species (2016): e.T6204A45225150. https://dx.doi.org/10.2305/IUCN.UK.2016-1.RLTS.T6204A45225150.en. Accessed on 06 October 2022.
*Halichoerusgrypus* (Fabricius, 1791)	least concern	Bowen D (2016). Halichoerusgrypus. The IUCN Red List of Threatened Species (2016): e.T9660A45226042. https://dx.doi.org/10.2305/IUCN.UK.2016-1.RLTS.T9660A45226042.en. Accessed on 06 October 2022.
